# Couch-Surfing and HIV Risk Behavior Among Young Adults Experiencing Homelessness

**DOI:** 10.1007/s10461-025-05009-x

**Published:** 2026-01-27

**Authors:** Laura Petry, Laura Onasch-Vera, Phebe Vayanos, Benjamin Henwood, Eric Rice

**Affiliations:** 1 University of Southern California Suzanne Dworak-Peck School of Social Work, California Los Angeles, USA; 2 University of Southern California Viterbi School of Engineering, California Los Angeles, USA; 3 University of California, Berkeley School of Public Health, California Berkeley, USA

**Keywords:** Homeless youth, Couch-surfing, Transactional sex, Social support, Egocentric network analysis

## Abstract

Young adults experiencing homelessness (YAEH) are significantly more likely to engage in HIV risk-related sexual behavior relative to their stably housed peers, and their social support networks can influence their engagement in these behaviors. However, few studies have investigated HIV risk behaviors among YAEH who are “couch-surfing,” a highly prevalent network-based survival strategy that involves cycling through temporary, informal housing arrangements. The current study utilizes survey data collected from 461 YAEH accessing drop-in center services in Los Angeles, California, between September 2016 and October 2018. Egocentric network analysis was used to examine associations among couch-surfing, sources of social support, and HIV risk and prevention behaviors. The potential moderating effect of social support on the relationship between couch-surfing and specific sexual risk behaviors was also tested. Compared to street- and shelter-based youth, couch-surfing YAEH reported the highest rates of recent transactional sex (18.0%) and concurrent or serial sexual partners (38.2%). Relative to residing in emergency shelter or transitional housing programs, couch-surfing was associated with over twice the odds of engaging in recent transactional sex (OR = 2.52, *p* = .023, 95% CI 1.13–5.62)—as was living unsheltered (OR = 2.06, *p* = .029, 95% CI 1.08–3.95). While social support was individually associated with several HIV risk-related sexual behaviors, its effect was ultimately eclipsed by homeless situation in the final model. Findings underscore the need for individual- and structural-level interventions that attend to the unique socioenvironmental contexts of couch-surfing YAEH.

## Introduction

Youth and young adults^1^ experiencing homelessness (YYAEH) face increased risk for multiple adverse health outcomes due to histories of abuse and victimization, unstable and frequently dangerous living situations, and engagement in behaviors often related to young people’s efforts to survive—including higher-risk substance use and sexual activity [1, 2]. Prior studies report an overall prevalence of sexually transmitted infections among YYAEH between 6 and 32% [3] and an HIV prevalence between 5 and 16% [4]. Yet even at its lowest estimate, the prevalence of HIV among YYAEH is at least twice as high as that of their stably housed peers [5]. HIV risk among YYAEH has been primarily attributed to higher-risk sexual behavior over illicit drug use, including condomless sex, sex under the influence of drugs or alcohol, multiple concurrent sex partners, and engagement in transactional sex (i.e., exchanging sex in return for money, drugs, shelter, or food) [6]. Central to understanding HIV risk-related sexual behavior among YYAEH are their social networks, or the web of interpersonal relationships surrounding an individual [6–9]. Social networks are also key to understanding young people’s homelessness trajectories, as YYAEH often engage in “couch-surfing,” a survival strategy whereby young people leverage personal social connections for temporary and informal housing arrangements [10, 11]. However, the extent to which couch-surfing may intersect with social support networks to affect HIV risk is largely unknown.

##  Literature Review

### HIV Risk-Related Sexual Behavior Among Youth and Young Adults Experiencing Homelessness

Decades of research have demonstrated that consistent and correct condom use is effective in reducing the risk of HIV transmission [12]. However, studies report that between 60 and 70% of YYAEH engage in condomless sex [6, 13]. Condomless sex is associated with other higher-risk sexual behaviors among this population, including sex under the influence, multiple concurrent partners, and transactional sex. Sex under the influence has been estimated to occur in one-third of sexual encounters reported by YYAEH [14]. Approximately 36% of YYAEH reporting on sexual activity over a 12-month period indicated engaging with multiple concurrent partners [8]. Reported rates of transactional sex among YYAEH range between 11 and 41% [15], with racial, ethnic, sexual, and gender minority youth and young adults found to be significantly more likely than their White, heterosexual, and cisgender counterparts to report engaging in transactional sex [15–18].

Routine HIV testing is a core strategy for helping to reduce transmission rates and facilitate early detection and treatment [19]. Overall HIV testing rates are higher among YYAEH than among the general youth and young adult population [20], with one representative sample of sexually active YYAEH in Los Angeles reporting an 85% lifetime test rate [5]. In addition to condom use and HIV testing, the use of pre-exposure prophylaxis (PrEP) is another key method for reducing transmission among high-risk groups [19]. However, awareness and uptake of PrEP among YYAEH remain low. Results from a cross-sectional survey of young adults experiencing homelessness across seven U.S. cities indicated that while 84% of young people were eligible for PrEP based on their risk behaviors, only 29% had any knowledge or awareness of the HIV prevention medication [13].

### The Influence of Social Support on HIV Risk-Related Sexual Behavior Among Youth and Young Adults Experiencing Homelessness

The influence of social networks in the lives of YYAEH is well-documented in the literature, including how key social connections can exacerbate or mitigate sexual risk behavior. Ecological models for understanding risk and resilience among YYAEH emphasize the particular importance of familial, peer, and social service relationships [21]. These relationships include young people’s families of origin, friends from home, other young people experiencing homelessness, intimate partners, and service provider staff. Relationships with supportive adults among this population have been connected to a reduced likelihood of condomless sex [22], while connections to stably housed peers have been associated with increased condom use [23]. Additionally, YYAEH affiliated with network members who have concurrent sexual partners are reported to be significantly more likely to have concurrent partners themselves [8]. Meanwhile, family relationships have been associated with decreased odds of engagement in transactional sex among YYAEH, while peers may have the opposite effect among LGBTQ+ youth and young adults [17]. However, more recent studies investigating the effects of social networks on the sexual health behavior of YYAEH largely focus on network structure and composition [6, 8, 17]. The more functional aspects of their networks, such as social support, are less often explored. 

*Social support* refers to either the perceived or actual resources available from the relationships surrounding a given individual [24]. Social support can take a variety of forms, of which the most common include emotional (affect, concern); instrumental (practical or material aid); informational (counsel, knowledge); and appraisal (feedback, affirmation). These varied forms of support are all intimately linked to an individual’s health and well-being, and evidence among higher-risk populations suggests that the presence *and* source of social support within an individual’s network are associated with both sexual risk behaviors and safer sex practices [25]. Although social support has been widely explored with other health-related outcomes among YYAEH, including mental health symptomatology and substance use [20–26], comparatively fewer have examined its effects on HIV risk-related sexual behavior.

### Couch-Surfing Among Youth and Young Adults Experiencing Homelessness

“Couch-surfing,” or moving from one temporary housing arrangement to another with no safe or stable alternative, is highly prevalent among YYAEH. Among the estimated 4.2 million YYAEH in the U.S. each year, over half report couch-surfing either exclusively or in combination with residing in shelter programs, living on the streets, or sleeping in other places not meant for human habitation [10, 27]. Meanwhile, youth and young adults who are couch-surfing are systematically excluded from accessing most housing resources available to individuals experiencing homelessness in the U.S. This is primarily due to narrow definitions of homelessness employed by the U.S. Department of Housing and Urban Development (HUD), which is the largest funder of housing resources available for those experiencing homelessness [28]. The definitional challenges surrounding efforts to serve YYAEH are long-standing and covered extensively elsewhere [29–31]. At its core, the de-prioritization and ultimate exclusion of couch-surfing youth and young adults from the mainstream homelessness response system is the result of presuming that couch-surfing is a safer experience than other forms of homelessness and therefore less worthy of intervention [29–32].

Despite its prevalence and its pertinence to policy responses to youth and young adult homelessness, couch-surfing remains a chronically understudied phenomenon. While it is broadly understood that youth and young adults often draw upon a broad range of social connections to facilitate temporary housing arrangements—including family, friends, intimate partners, and strangers—the structure of these networks and their influence on young people’s health, safety, and well-being is less understood [11, 33]. A burgeoning body of research suggests that couch-surfing may be specifically associated with a range of vulnerabilities related to mental health, substance use, and discrimination [34–36], but its relationship to HIV risk warrants further investigation.

### Couch-Surfing, Social Support, and HIV Risk-Related Sexual Behavior Among Youth and Young Adults Experiencing Homelessness

Evidence suggests that specific living situations and contexts may have distinct associations with sexual health behavior [37], but how this transpires among YYAEH is relatively underexplored—and altogether unknown for those who are couch-surfing. Earlier work has indicated that youth and young adults living on the streets are significantly less likely to use condoms and those residing in emergency shelters are more likely to report a greater number of sexual partners [38]. Transactional sex has also been noted to occur at lower rates among youth and young adults in shelters compared to those living on the streets [15]. One study of transgender and gender-expansive adults indicated that those who were couch-surfing or in other temporary living arrangements were twice as likely to report engaging in transactional sex relative to those who had not sought a temporary place to stay [39].

While there is evidence pointing toward differential associations between specific homeless situations and HIV risk-related sexual behaviors among young adults, the unique socioenvironmental contexts of couch-surfing remain understudied. Although it is possible that some couch-surfing arrangements can include exposure to supportive adults and peers who might mitigate risk, others could involve engaging network members that facilitate or otherwise influence higher-risk behavior (e.g., exchanging sex in return for a place to stay). Consequently, the potential impact of couch-surfing on HIV risk-related sexual behaviors among young adults may in part depend on the composition of their social support networks.

## Current Study

Despite extensive research on HIV risk among YYAEH, scant studies have examined the extent to which the socioenvironmental contexts of couch-surfing may be associated with specific sexual behaviors. Similarly, while social network influences on the sexual risk-taking of YYAEH have been widely explored, fewer studies investigate how the functional aspects of these networks (e.g., the provision of social support) may be associated with risk among this population. To help address these gaps in the literature, the current study uses cross-sectional data to conduct an egocentric social network analysis to explore associations among couch-surfing, sources of social support, and HIV risk-related sexual behaviors among young adults experiencing homelessness (YAEH). Additionally, given that couch-surfing is a temporary housing strategy that hinges on a young person’s ability to leverage their social networks, the current study also aims to examine how different sources of social support may moderate the association between couch-surfing and HIV risk-related sexual behavior. This study is guided by the following research questions and hypotheses:

- *Research Question 1*: Is couch-surfing specifically associated with engagement in HIV risk-related sexual behavior among young adults experiencing homelessness?
  **Hypothesis 1** Young adults who are couch-surfing will be at significantly greater odds of engaging in transactional sex relative to those residing in emergency shelter or transitional housing programs.
- *Research Question 2*: Are specific sources of social support associated with engagement in HIV risk-related sexual behavior among young adults experiencing homelessness?
  **Hypothesis 2** Young adults receiving social support from family members will be at significantly lesser odds of reporting condomless sex, concurrent or serial partners, sex under the influence, and transactional sex.
  **Hypothesis 3** Young adults receiving social support from service provider staff will be at significantly greater odds of reporting recent HIV testing and PrEP knowledge and use.
- *Research Question 3*: Does social support moderate the relationship between couch-surfing and HIV risk-related sexual behavior among young adults experiencing homelessness?
  **Hypothesis 4** The association between couch-surfing and HIV risk-related sexual behavior will be moderated by social support, such that supportive connections to family will weaken the relationship between couch-surfing and higher-risk behaviors. In particular, supportive family relationships will weaken the relationship between couch-surfing and transactional sex.

## Methods

### Participants and Procedures

This study utilized baseline data from *Have You Heard?*, a longitudinal study of a peer-led social network intervention to prevent HIV among YYAEH [40]. Data were collected via convenience sampling methods across three drop-in centers serving YYAEH in Los Angeles, California, between September 2016 and October 2018. All youth and young adults accessing services from the partner agencies were eligible for the study and approached by research staff upon entering a given drop-in center.

At the time of study enrollment, each participant provided informed consent and was assigned a unique identifier to ensure their anonymity throughout the study. Contact information was collected for the sole purpose of reaching participants for follow-up surveys. This information was maintained separately from informed consent forms and survey instruments and destroyed at the conclusion of the study. Across three waves of data collection, each participant completed a computerized, self-administered survey approximately 60 minutes in length. Survey items included an assessment of the young person’s sociodemographic characteristics, personal social networks, and HIV-related risk behaviors. All survey items had been successfully utilized in prior research with similar study populations in drop-in center settings [41, 42]. Participants were compensated for their time with a $20 gift card at baseline, $25 gift card at the one-month follow-up, and $30 gift card at the three-month follow-up. Study procedures and survey items were approved by the University of Southern California Institutional Review Board.

In the current study, only baseline data were analyzed due to the level of attrition between baseline and subsequent waves resulting in small cell sizes across the main variables of interest. A total of 731 youth and young adults between the ages of 14 and 26 participated in the parent study at baseline. Given the distinct developmental considerations for youth under the age of 18 and young adults between 18 and 25 years old [43], participants younger than 18 (*n* = 14) or older than 25 (*n* = 6) were removed from the dataset. Additionally, participants who reported spending most of their nights somewhere other than in a shelter program, on the streets, or couch-surfing were also removed. This included young adults staying in their own place (*n* = 73), in a hotel or motel (*n* = 37), in an institutional setting (e.g., foster care, jail or prison, or inpatient facility; *n* = 4), or someplace else (*n* = 35). While these other living situations may also hold unique bearings on young people’s social support networks and risk behaviors, they are distinct experiences outside the scope of the current study. These exclusions resulted in 556 young adults between the ages of 18 and 25 in the final analytic dataset.

### Measures

#### Control Variables

##### Demographic Characteristics

*Age*,* gender identity*,* sexual orientation*, and *race or ethnicity* were included as control variables in the current analysis. *Age* was a continuous variable inclusive of ages between 18 and 25. *Gender identity* was a nominal variable that included male, female, transgender, and gender-expansive. Due to the relatively small numbers in the latter group, those who were non-binary, genderqueer, gender non-conforming, or another gender-expansive identity (*n* = 23) were combined with transgender participants into a single category. Male participants were used as the referent group. *Sexual orientation* was a dichotomous variable in which a value of zero denoted heterosexual participants and a value of one represented lesbian, gay, bisexual, questioning, asexual, or other sexual minority (LGBQ+) participants. *Race or ethnicity* was a nominal variable with four categories: Black, Hispanic/Latinx, White, and either multiracial or some other race or ethnicity. Due to small cell sizes across the different forms of homelessness under study, participants reporting some other race or ethnicity were combined with those identifying as multiracial. This included young adults who were American Indian or Alaska Native (*n* = 18), Asian (*n* = 8), and Native Hawaiian or Other Pacific Islander (*n* = 1). White participants were used as the referent group.

#### Independent Variables

##### Current Homeless Situation

The present study sought to examine the relationship between different forms of homelessness and social support networks, and their association with various HIV risk-related sexual behaviors. Current homeless situation was a nominal variable that distinguished between *couch-surfing*,* unsheltered*, and *sheltered* young adults. These situations were originally assessed by asking participants to identify the type of location they spent most of their nights over the preceding two weeks. Participants who reported staying at the home of someone they knew (e.g., family member, friend, or romantic partner) or with a stranger were coded as *couch-surfing*.^2^ Participants who indicated living outside or in a tent, vehicle, abandoned building, or similar location were coded as being *unsheltered*. Those who reported residing in either an emergency shelter or transitional housing program were coded as being *sheltered*. Sheltered participants were used as the referent group, providing a point of comparison between young adults temporarily residing inside private residences and those temporarily residing inside social service facilities.

##### Sources of Social Support

Social support variables were derived from survey items designed to identify the different types of support provided by different types of individuals within a young person’s network [44]. The parent study adhered to recommendations for incorporating egocentric network analysis into social work research [45]; participants were asked to name up to five individuals with whom they had the most contact in the past month, and to then identify which individuals held specific characteristics and provided specific kinds of support. In the current study, five key types of social network members were examined: *family*, *home-based peers*,* romantic or sexual partners*,* homeless peers*, and *service provider staff.* The selection of these network member types was based on empirical evidence regarding the positive and negative influences of specific familial, peer, and social service relationships on YYAEH [21].

*Family* included biological, adopted, or foster family members, and excluded street or other chosen family. *Home-based peers* included housed peers between the ages of 14 and 25 whom the participant knew prior to experiencing homeless. *Romantic or sexual partners* included individuals with whom participants reported being romantically or sexually involved. *Homeless peers* included other young people between the ages of 14 and 25 who were also currently experiencing homelessness. *Service provider staff* included individuals the participant identified as a case manager, service provider staff member, or agency volunteer.

Social support was measured by asking participants to identify which individuals in their named network provided either *emotional*,* instrumental*,* informational*, or *appraisal* support in the past month. *Emotional support* was assessed by asking who participants had turned to for help or advice when they were feeling depressed or dealing with major issues. *Instrumental support* was assessed by asking who participants were able to borrow money or other material aid from when needed. *Informational support* was assessed by asking who participants had spoken to about where to get social services. *Appraisal support* was assessed by asking who encouraged participants in meeting their goals.

Although both the *source* and *type* of social support provided can affect individual outcomes [46], small cell sizes observed in the current study precluded such disaggregation. Consequently, the endorsement of at least one form of support was used to create a dichotomous variable representing whether a given network member provided social support. Together with the network member variables, this broader social support variable was used to construct a series of dichotomous variables indicating the presence of specific supportive relationships within a young person’s network. For example, young adults who reported having at least one family member who provided social support were coded as having social support from family. These variables were subsequently integrated with each participant’s individual-level survey data.

#### Dependent Variables

##### HIV Risk-Related Sexual Behaviors

Participants were asked a series of questions about their sexual behavior over the past 30 days based on whether they indicated having at least one sexual partner during that time. Similar to how their broader social networks were assessed, participants identified up to five recent sexual partners and were asked whether they engaged in specific sexual practices with a given partner. *Condomless anal sex*, *condomless vaginal sex*, *concurrent or serial sexual partners*,* sex under the influence*, and *transactional sex* were created as dichotomous variables for inclusion in the current analysis. Participants were coded as having *condomless anal sex* if they identified at least one sexual partner with whom they had anal sex without a condom; *condomless vaginal sex* was coded similarly. *Concurrent or serial sexual partners* was coded to indicate whether participants reported having two or more sexual partners in the past 30 days. *Sex under the influence* was coded according to whether participants identified at least one sexual partner with whom they had sex while either themselves or their partner were under the influence of drugs or alcohol. Finally, *transactional sex* was derived from items measuring both recent exchange and survival sex. Exchange sex was defined as trading sex or sexual content (i.e., videos or photos) in exchange for money, while survival sex was defined as trading sex or sexual content in exchange for any material support other than money, including a place to stay. Participants who reported being sexually inactive were coded as not engaging in any of these HIV risk-related sexual behaviors.

All participants, regardless of whether they were sexually active, were asked about their HIV prevention behaviors. *HIV testing*, *PrEP knowledge*, and *ever being prescribed PrEP* were created as dichotomous variables for inclusion in the current analysis. *HIV testing* was coded to reflect participants who reported testing for HIV within the previous six months. *PrEP knowledge* was assessed using a Likert scale to measure how much participants knew about PrEP; participants who expressed having at least “a little bit” of knowledge about the HIV prevention medication were coded as having PrEP knowledge. *Ever being prescribed PrEP* was a dichotomous variable indicating whether participants reported ever having a PrEP prescription.

### Analysis

Egocentric network analysis is a type of social network analysis facilitating the statistical examination of the social connections surrounding a single individual (the “ego”) [47]. This type of analysis enables researchers to examine not only the diversity of these network connections, but the ways in which specific relationships influence individual attitudes and behaviors [45]. Further, egocentric network analysis provides a method by which personal network characteristics can be considered across different attributes held by the ego—and how these factors coalesce to influence access to information, resources, status, and social support [45, 48]. Given the importance of various relationships in the lives of YAEH, including their role in facilitating couch-surfing arrangements, egocentric network analysis is well suited for exploring how certain facets of these relationships may be differentially associated with engagement in HIV risk-related sexual behaviors.

Descriptive statistics of HIV risk-related sexual behaviors and sources of social support across the different forms of homelessness under study were first run to examine their frequency among the current sample. Chi-square tests were then performed to help identify statistically significant relationships (defined as *p* < .10) between young people’s current homeless situation, sources of social support, and various HIV risk-related sexual behaviors. Statistically significant behaviors were carried forward into a series of bivariate logistic regressions to examine the directionality and strength of their associations with couch-surfing and sources of social support. Significant sources of social support were also examined in a series of logistic regressions testing the potential moderating effect of social support on the association between couch-surfing and HIV risk-related sexual behavior. In these bivariate analyses, a *p-*value threshold of 0.1 was used to determine which variables were candidates for further investigation and for inclusion in multivariable logistic regression models. Final multivariable models included HIV risk-related sexual behaviors that were statistically significant at the level of *p* < .05 among couch-surfing young adults, sources of social support that were significant in relationship to those particular behaviors in the preceding bivariate analysis, and demographic control variables.

## Results

Table 1 presents the demographic characteristics of the current sample overall and by homeless situation. The average age of participants was 21.9 years old. The majority of participants were young people of color—31.5% were Black, 14.5% were Hispanic/Latinx, and 30.9% were multiracial or another non-White race or ethnicity. Over half (66.9%) of young adults were male, 21.4% were female, and 11.7% were transgender or gender-expansive. A sizeable minority (42.6%) identified as LGBQ+. Most young adults reported currently living unsheltered (53.6%), while 16.0% were couch-surfing and 30.4% were residing in an emergency shelter or transitional housing program. Across the different forms of homelessness under study, young adults differed significantly with respect to age *F*(2,553) = 5.21, *p* = .006; race and ethnicity, *X*^*2*^ (6, *N =* 553) = 17.96, *p* = .006; and sexual orientation, *X*^*2*^ (2, *N =* 550) = 7.0, *p* = .030. Post hoc analyses indicated that young adults who were couch-surfing had a significantly lower average age compared to young adults who were unsheltered (*p* = .004). Meanwhile, higher rates of Black young adults were observed among those couch-surfing or in shelter (37.1% and 39.5%, respectively) compared to those who were unsheltered (29.0%). Higher rates of White young adults were observed among the unsheltered (29.0%) compared to those couch-surfing (19.1%) or in shelter (15.0%). Young adults did not significantly differ in gender identity with respect to their current homeless situation.

**Table 1:** Characteristics of study participants, by homeless situation (*n* = 556)

	Couch-surfing (*n* = 89) *n* (%)	Unsheltered (*n* = 298) *n* (%)	Sheltered (*n* = 169) *n* (%)	Total (*n* = 556) *n* (%)	F or X^2^
Demographics					
Age, mean (SD)	21.4 (2.1)	22.1 (2.0)	21.9 (1.9)	21.9 (2.0)	**5.21**
Race and ethnicity (*n* = 553)					**17.96**
Black	33 (37.1)	75 (29.0)	66 (39.5)	174 (31.5)	
Hispanic/Latinx	13 (14.6)	44 (14.8)	23 (13.8)	80 (14.5)	
White	17 (19.1)	86 (29.0)	25 (15.0)	128 (23.2)	
Multiracial or another race or ethnicity	26 (29.2)	92 (31.0)	53 (31.7)	171 (30.9)	
Gender identity					3.37
Male	61 (68.5)	203 (68.1)	108 (63.9)	372 (66.9)	
Female	20 (22.5)	64 (21.5)	35 (20.7)	119 (21.4)	
Transgender or gender-expansive	8 (9.0)	31 (10.4)	26 (15.4)	65 (11.7)	
LGBQ+ (*n* = 550)	33 (37.1)	116 (39.5)	85 (50.9)	234 (42.6)	**7.00**

Numbers in bold type indicate significance at *p* < .1

Table 2 displays the frequencies of specific HIV risk and prevention behaviors and sources of social support across the different forms of homelessness under study. At 18.0%, couch-surfing young adults reported the highest rate of recent transactional sex, compared to 9.5% of sheltered and 15.4% of unsheltered young adults. The results of a chi-square test indicated that young adults engaging in transactional sex and those who did not differed significantly with respect to their current homeless situation, *X*^2^ (2, *N =* 556) = 4.56, *p* = .093. The rate of concurrent or serial sexual partners was also highest among couch-surfing young adults, with 38.2% reporting multiple partners compared to 32.0% of sheltered and 34.9% of unsheltered young adults.

**Table 2:** HIV risk and prevention behaviors and sources of social support among young adults, by current homeless situation (*n* = 556)

	Couch-surfing (*n* = 89) *n* (%)	Unsheltered (*n* = 298) *n* (%)	Sheltered (*n* = 169) *n* (%)	X^2^
HIV risk behavior (past 30 days)				
Condomless anal sex	12 (13.5)	79 (26.5)	40 (23.7)	**6.46**
Condomless vaginal sex	26 (29.2)	103 (34.6)	40 (23.7)	**6.12**
Concurrent or serial partners	34 (38.2)	104 (34.9)	54 (32.0)	1.05
Sex under the influence	16 (18.0)	92 (30.9)	28 (16.6)	**14.35**
Transactional sex	16 (18.0)	46 (15.4)	16 (9.5)	**4.56**
HIV prevention behavior				
HIV test in past 6 months (*n* = 550)	66 (75.0)	204 (68.9)	129 (77.7)	4.44
PrEP knowledge (*n* = 548)	42 (47.2)	117 (39.9)	86 (51.8)	**6.31**
Ever prescribed PrEP	10 (11.2)	25 (8.4)	15 (8.9)	0.68
Source of social support				
Homeless peer	31 (34.8)	142 (47.7)	85 (50.3)	**6.01**
Home-based peer	30 (33.7)	54 (18.1)	41 (24.3)	**9.99**
Romantic/sexual partner	33 (37.1)	114 (38.3)	54 (32.0)	1.90
Family	41 (46.1)	118 (39.6)	61 (36.1)	2.43
Service provider staff	21 (23.6)	61 (20.5)	49 (29.0)	4.35

Numbers in bold type indicate significance at *p* < .1

Couch-surfing young adults reported the lowest rate of condomless anal sex (13.5%) relative to both their sheltered (23.7%) and unsheltered (26.5%) counterparts. Results of a Chi-square test indicated that young adults who reported condomless anal sex and those who did not differed significantly with respect to their current homeless situation, *X*^2^ (2, *N =* 556) = 6.46, *p* = .040. Meanwhile, 29.2% of couch-surfers reported engaging in condomless vaginal sex, a rate higher than young adults in shelter but lower than those living unsheltered (23.7% and 34.6%, respectively).

Sex under the influence occurred at similar rates among couch-surfing young adults and young adults in shelter (18.0% and 16.6%, respectively) and was highest among those who were unsheltered (30.9%). The results of a chi-square test indicated that young adults reporting having sex under the influence and those who did not differed significantly with respect to their current homeless situation, *X*^2^ (2, *N =* 556) = 14.35, *p* = .001.

While the overall majority of young adults reported testing for HIV within the past six months, the rate was highest among young adults in shelter (77.7%), followed by those who were couch-surfing (75.0%) and unsheltered (68.9%). Knowledge of PrEP followed a similar pattern, though overall rates were lower than for HIV testing. Just over half (51.8%) of young adults in shelter reported having at least some knowledge of the HIV prevention medication, followed by 47.2% of couch-surfers and 39.9% of unsheltered young adults. The results of a chi-square test indicated that young adults reporting knowledge of PrEP and those who did not differed significantly with respect to their current homeless situation, *X*^2^ (2, *N =* 548) = 6.31, *p* = .043. Rates of ever being prescribed PrEP were low across the board, with 11.2% of couch-surfing young adults, 8.9% of sheltered young adults, and 8.4% of unsheltered young adults reporting ever having a prescription for the HIV prevention medication.

Sources of social support also differed according to young people’s homeless situation. Couch-surfing young adults most frequently reported receiving social support from family (46.1%) and home-based peers (33.7%). Meanwhile, support from homeless peers was most frequently cited by both unsheltered and sheltered young adults (47.7% and 50.3%, respectively). Couch-surfing and unsheltered young adults reported similar rates of receiving support from a romantic or sexual partner (37.1% and 38.3%, respectively). Young adults in shelter reported the highest rate of support from service provider staff (29.0%). Chi-square tests indicated that young adults reporting supportive homeless peers and those who did not differed significantly based on their current homeless situation, *X*^2^ (2, *N =* 556) = 6.01, *p* = .050, as did those who reported supportive home-based peers and those who did not, *X*^2^ (2, *N =* 556) = 9.99, *p* = .007.

Table 3 displays the bivariate associations between current homeless situation, sources of social support, and HIV risk and prevention behaviors that were significant in the preceding analysis: condomless anal sex, condomless vaginal sex, sex under the influence, transactional sex, and PrEP knowledge. Relative to residing in a shelter, couch-surfing was significantly associated with decreased odds of condomless anal sex (OR = 0.50, *p =* .056, 95% CI 0.25–1.02) and increased odds of recent transactional sex (OR = 2.10, *p =* .052, 95% CI 0.98–4.42). Living unsheltered was significantly associated with increased odds of condomless anal sex (OR = 1.16, *p* = .499, 95% CI 0.75–1.80); condomless vaginal sex (OR = 1.70, *p* = .015, 95% CI 1.11 = 2.61); sex under the influence (OR = 2.25, *p* = .001, 95% CI 1.40–3.61); and transactional sex (OR = 1.75, *p* = .070, 95% CI 0.95–3.19). Living unsheltered was also significantly associated with decreased odds of knowledge about PrEP (OR = 0.62, *p* = .014, 95% CI 0.42–0.91).

**Table 3:** Bivariate analysis of odds of engaging in HIV risk-related sexual behaviors among young adults experiencing homelessness

	Condomless anal sex	Condomless vaginal sex	Sex under the influence	Transactional sex	PrEP knowledge
	OR (95% CI)	OR (95% CI)	OR (95% CI)	OR (95% CI)	OR (95% CI)
Homeless situation *(ref. Sheltered)*					
Couch-surfing	**0.50 (0.25–1.02)**	1.33 (0.75–2.37)	1.10 (0.56–2.17)	**2.10 (0.98–4.42)**	0.83 (0.50–1.39)
Unsheltered	**(0.75–1.80)**1.16	**1.70 (1.11–2.61)**	**2.25 (1.40–3.61)**	**1.75 (0.95–3.19)**	**0.62 (0.42–0.91)**
Source of social support					
Homeless peer	**1.57 (1.06–2.33)**	0.95 (0.66–1.37)	**1.59 (1.08–2.35)**	**0.64 (0.39–1.05)**	**1.39 (0.99–1.94)**
Home-based peer	1.09 (0.69–1.74)	1.21 (0.79–1.85)	1.34 (0.86–2.09)	**0.59 (0.31–1.13)**	0.81 (0.54–1.22)
Romantic or sexual partner	**3.94 (2.62–5.94)**	**2.54 (1.75–3.69)**	**2.34 (1.58–3.47)**	0.99 (0.60–1.63)	**1.38 (0.97–1.95)**
Family	0.97 (0.65–1.44)	1.16 (0.80–1.67)	1.05 (0.71–1.56)	**0.64 (0.38–1.07)**	1.05 (0.74–1.48)
Service provider staff	1.32 (0.84–2.06)	0.87 (0.57–1.35)	1.30 (0.83–2.02)	0.81 (0.45–1.47)	**1.62 (1.09–2.41)**

Numbers in bold type indicate significance at *p* < .1

 Supportive ties to homeless peers were associated with increased odds of condomless anal sex (OR = 1.57, *p =* .025, 95% CI 1.06–2.33), sex under the influence (OR = 1.59, *p* = .019, 95% CI 1.08–2.35) and knowledge about PrEP (OR = 1.39, *p* = .059, 95% CI 0.99–1.94). Support from romantic or sexual partners echoed these trends and was additionally associated with increased odds of condomless vaginal sex (OR = 2.54, *p* < .001, 95% CI 1.75–3.69). Decreased odds of recent transactional sex were associated with support from homeless peers (OR = 0.64, *p* = .080, 95% CI 0.39–1.05), home-based peers (OR = 0.59, *p* = .091, 95% CI 0.31–1.13) and family (OR = 0.64, *p* = .088, 95% CI 0.38–1.07). Support from service provider staff was associated with increased odds of reporting any knowledge of PrEP (OR = 1.62, *p =* .017, 95% CI 1.09–2.41).

Tables 4 and 5 examine whether sources of social support moderate the association between couch-surfing and specific HIV risk-related sexual behaviors. From the preceding bivariate analysis, only behaviors significant at the level of *p* < .10 among couch-surfing young adults were examined, as were sources of social support that were significant in relationship to this subset of behaviors at the level of *p* < .10. Results indicated no significant interaction effect between current homeless situation and social support with respect to either condomless anal sex or recent transactional sex. Consequently, interaction terms were excluded from the final multivariable models.

**Table 4:** Interaction effects between social support and current homeless situation and their association with recent condomless sex (past 30 days)

	Condomless anal sex OR (95% CI)
Support from homeless peer	
Couch-surfing *(ref. Sheltered)*	0.68 (0.27–1.71)
Unsheltered *(ref. Sheltered)*	1.23 (0.63–2.38)
Homeless peer support	1.67 (0.81–3.43)
Couch-surfing × homeless peer support	0.55 (0.13–2.42)
Unsheltered × homeless peer support	0.93 (0.38–2.25)
Support from romantic or sexual partner	
Couch-surfing *(ref. Sheltered)*	0.74 (0.27–2.01)
Unsheltered *(ref. Sheltered)*	1.16 (0.60–2.24)
Romantic or sexual partner support	4.95 (2.33–10.51)
Couch-surfing × romantic or sexual partner support	0.37 (0.09–1.58)
Unsheltered × romantic or sexual partner support	0.84 (0.33–2.13)

**Table 5:** Interaction effects between social support and homeless situation and their association with recent transactional sex (past 30 days)

	Transactional sex OR (95% CI)
Support from homeless peer	
Couch-surfing *(ref. Sheltered)*	1.73 (0.68–4.39)
Unsheltered *(ref. Sheltered)*	1.62 (0.74–3.52)
Homeless peer support	0.56 (0.19–1.62)
Couch-surfing × homeless peer support	1.46 (0.30–7.04)
Unsheltered × homeless peer support	1.18 (0.34–4.08)
Support from home-based peer	
Couch-surfing *(ref. Sheltered)*	2.50 (1.08–5.80)
Unsheltered *(ref. Sheltered)*	1.73 (0.89–3.38)
Home-based peer support	0.70 (0.19–5.80)
Couch-surfing × home-based peer support	0.56 (0.09–3.67)
Unsheltered × home-based peer support	0.91 (0.19–4.50)
Support from family	
Couch-surfing *(ref. Shelter)*	2.62 (1.05–6.56)
Unsheltered *(ref. Shelter)*	1.91 (0.92–3.96)
Family support	0.79 (0.26–2.38)
Couch-surfing × family support	0.59 (0.12–2.93)
Unsheltered × family support	0.79 (0.26–2.38)

Table 6 features the final model containing significant results for couch-surfing young adults. Couch-surfing was associated with over twice the odds of reporting recent transactional sex relative to residing in shelter (OR = 2.52, *p =* .023, 95% CI 1.13–5.62), as was living unsheltered (OR = 2.06, *p =* .029, 95% CI 1.08–3.95). Relative to their White peers, Hispanic/Latinx young adults indicated over twice of the odds of reporting recent transactional sex (OR = 2.35, *p =* .025, 95% CI 1.11–4.98), as did LGBQ + young adults relative to their heterosexual counterparts (OR = 2.18, *p* = .005, 95% CI 1.26–3.76). None of the social support variables—support from homeless peers, home-based peers, or family members—were statistically significant in the final model.

**Table 6:** Multivariable logistic regression model of factors associated with recent transactional sex (past 30 days) among young adults experiencing homelessness

	Transactional sex OR (95% CI)
Homeless situation *(ref. Sheltered)*	
Couch-surfing	2.52 (1.13–5.62)*
Unsheltered	2.06 (1.08–3.95)*
Source of social support	
Homeless peer	0.62 (0.36–1.05)
Home-based peer	0.61 (0.30–1.22)
Family	0.76 (0.43–1.32)
Demographics	
Age	1.05 (0.93–1.20)
Race and ethnicity *(ref. White)*	
Black	1.11 (0.54–2.28)
Hispanic/Latinx	2.35 (1.11–4.98)*
Multiracial or another race or ethnicity	0.82 (0.40–1.69)
Gender identity *(ref. Male)*	
Female	1.09 (0.58–2.04)
Transgender or gender-expansive	1.65 (0.78–3.48)
LGBQ+	2.18 (1.26–3.76)*

*p < .05

## Discussion

Current study findings provide new evidence for vulnerabilities associated with couch-surfing among YAEH. Relative to those in shelter, couch-surfing young adults were over twice as likely to engage in recent transactional sex (OR = 2.52, *p* = .023, 95% CI 1.13–5.62), as were unsheltered young adults (OR = 2.06, *p* = .029, 95% CI 1.08–3.95). These findings not only suggest a distinct association between specific forms of homelessness and young people’s recent engagement in transactional sex, but that the magnitude of this relationship is similar between couch-surfers and unsheltered young adults. Transactional sex places young people at increased risk for HIV [16, 46], and its association with couch-surfing—a highly prevalent but largely hidden and understudied form of homelessness—underscores the need for more context-specific approaches to reducing HIV risk-related sexual behavior among YAEH. Although the degree to which couch-surfing young adults in this sample are residing with the people with whom they are transacting sex, findings echo prior work suggesting that transactional sex with a casual partner is associated with housing instability [49]. While we are unable to disaggregate the different types of couch-surfing hosts in this dataset, it is possible that some young people’s couch-surfing arrangements are intertwined with and contingent upon transactional sex, particularly in scenarios where intimate partners or strangers are hosting. Given that couch-surfing hosts may also include supportive family and friends—relationships with whom young people feel especially protective [11, 36]—we must also consider transactional sex as a tactic employed by young adults to avoid ‘overstaying their welcome’ with a given host while also avoiding the streets and the shelter system. Independent of these nuances, current findings challenge assumptions that couch-surfing is an inherently safer or less vulnerable experience than other forms of homelessness and highlight the need for more nuanced policy responses attending to the realities of youth and young adult homelessness.

Notably, Hispanic/Latinx young adults were also over twice as likely to report engaging in recent transactional sex relative to their White peers (OR = 2.35, *p* =.025, 95% CI 1.11–4.98). While one previous study on transgender and gender-expansive adults experiencing homelessness indicated that Black, Hispanic/Latinx, and multiracial adults were more likely to report engaging in transactional sex [38], studies drawing from either nationwide samples of YAEH or youth and young adults more broadly have only reported Black youth and young adults as more likely to engage in transactional sex [15, 50]. However, these studies also contrast with earlier work, which often produced mixed findings on the association between race or ethnicity and transactional sex. Many of these studies worked with comparatively smaller, more localized samples of homeless youth populations [51–53], and current study findings could be considered within the context of rising Hispanic/Latinx homelessness in Los Angeles [54]. This general inconsistency in the literature warrants further investigation into potential qualitative differences that may vary across geographic contexts, intersecting social identities, and structural factors affecting engagement in transactional sex among racially and ethnically minoritized YAEH [15, 55]. In contrast, study findings that LGBQ + young adults were significantly more likely to report recent transactional sex relative to their heterosexual counterparts (OR = 2.18, *p* =.005, 95% CI 1.26–3.76) are consistent with previous literature establishing that sexual minority young adults are at increased risk of engaging in transactional sex [15, 18]. This may be attributed in part to the increased prevalence of other correlates of transactional sex observed among LGBQ + youth and young adults, including prior sexual victimization, less family support, and more connections to peers engaged in trading sex [56].

Beyond transactional sex, study findings provide important insights into several other HIV risk and prevention behaviors among couch-surfing young adults. Across the five HIV risk-related behaviors under study—condomless anal and vaginal sex, sex under the influence of drugs or alcohol, concurrent or serial sexual partners, and engaging in transactional sex—couch-surfing young adults reported the highest rates of both transactional sex (18.0%) and concurrent or serial sexual partners (38.2%). While couch-surfing young adults reported the lowest rate of condomless anal sex across all forms of homelessness under study (13.5%), their rates of condomless vaginal sex (29.2%) and sex under the influence (18.0%) were generally higher than those of young adults in shelter programs and lower than those who were unsheltered. These findings indicate that even when temporarily residing with others, YAEH still contend with elevated HIV risk.

Consistent with prior research [5], most YAEH in the overall sample reported testing for HIV within the preceding six months. The prevalence of testing among couch-surfing young adults (75.0%) was on par with the rate of testing observed among young adults in shelter (77.7%) and higher than the testing rate noted among unsheltered young adults (68.9%). PrEP knowledge followed a similar pattern, though rates were markedly lower than those for HIV testing. The particularly high rate of HIV testing observed among young adults in shelter echoes earlier work finding shelter use to be associated with increased HIV testing among YYAEH due to their more immediate proximity to services and linkages [57]. The lower rates of PrEP knowledge across the board are also consistent with research on the low awareness of PrEP among this population [13], as young adults in shelter reported the highest rates of PrEP knowledge (51.8%), followed by those who were couch-surfing (47.2%) and unsheltered (39.9%). The higher prevalence of PrEP knowledge among sheltered young adults is once again likely due to their proximity to social services, as service providers in these settings may leverage their physical and relational proximity to young people to facilitate access to HIV prevention information, resources, and services [58, 59].

In addition to the relationship between couch-surfing and HIV risk-related sexual behavior among YAEH, the extent to which social support may impact the likelihood of specific sexual behaviors—and whether social support moderates the relationship between couch-surfing and sexual risk—was of key interest. However, social support alone was only significant in the bivariate analysis; there was no significant interaction effect and no significant main effect of social support on risk observed in the multivariable models for transactional sex. These findings suggest that while different forms of homelessness and social support may be individually associated with transactional sex, the effect of a young person’s current homeless situation on engagement in transactional sex ultimately eclipses that of social support. Moreover, broad social support may have no bearing on the relationship between different forms of homelessness and transactional sex. However, prior work has indicated that it is not only the source of social support but the *type* of support provided that can vary across different network members and affect health outcomes [60]. While the current study was limited in its ability to disaggregate both sources and types of support, future research ought to investigate how these two facets of social support might influence HIV risk-related behaviors among YAEH. Emotional support might be particularly pertinent to engagement in risk behaviors among YAEH [26], and perceptions of both emotional and instrumental support may intersect to influence young people’s engagement in transactional sex [61].

Altogether, study findings concerning HIV risk-related sexual behavior and varied forms of homelessness among young adults suggest that the specific *context* of young people’s experiences of homelessness may coincide with different types of sexual risk behavior and warrant more targeted intervention strategies. While couch-surfing young adults reported the highest rates of transactional sex and concurrent or serial sexual partners, they also indicated higher rates of condom use and lower rates of sex under the influence relative to their peers living on the streets. Meanwhile, unsheltered young adults reported sex under the influence at nearly twice the rate of their peers who were couch-surfing or in shelter. Overall, young adults living on the streets in this sample appeared to experience a higher level of sexual risk while young adults in shelter indicated higher engagement in HIV prevention behaviors. This aligns with earlier research indicating that living on the streets is associated with increased sexual risk-taking among youth and young adults [15, 62, 63], and that emergency shelter and transitional housing programs can serve as a protective factor against engagement in higher-risk sexual behavior [58, 59]. These findings also point toward how profiles of sexual risk among young adults may differ across homeless situations, and signal opportunities to tailor HIV prevention strategies to the specific environmental contexts of young people’s experiences of homelessness. As established earlier, the literature on youth and young adult homelessness seldom engages comparisons across different forms of homelessness, but current findings underscore the importance of disaggregating these homelessness situations to better understand how to reduce HIV risk in this population.

## Limitations and Future Research

A number of limitations to the current study warrant acknowledgment. Given the sensitive nature of asking YAEH to share details on their sexual activity, social desirability bias may have impacted survey responses and subsequent study results. Additionally, the cross-sectional data utilized in the current analysis precluded the study from investigating any temporal relationships or drawing any causal interpretations. Longitudinal work is needed to examine how the relative stability of couch-surfing arrangements and social support networks over time may impact young people’s engagement in HIV-related sexual risk behavior. Of particular interest is whether specific changes in a young person’s couch-surfing status, their relationship to their couch-surfing host, or their overall support networks result in changes to the likelihood that they engage in transactional sex over time. Measuring the temporal, relational, and spatial dimensions of transactional sex encounters in relationship to a young person’s homeless situation is especially important. Future research should assess whether these encounters take place before, during, or after a specific couch-surfing arrangement; with a couch-surfing host or someone else within the same residence; and at the place they are temporarily residing or elsewhere.

The operationalization of social support in the current study is limited in its reliance on a broader measure of social support. To disaggregate the *types* of social support across network members (e.g., emotional, instrumental, informational, and appraisal support), future research is needed to assess the social support networks within a larger sample of couch-surfing young adults. Additionally, while the characterization of network member types in the parent study was grounded in the literature on the social networks of YAEH, researchers may consider additional network member types that may be relevant to specific phenomena under study. For example, recent research on couch-surfing among queer YAEH in Australia indicated that young people more frequently described experiencing coercive sexual exchange with peers within their own age group as opposed to older adults [64]. The current study was only able to broadly examine participants’ relationships with other young people due to the limited information collected on age in the original social network survey; no demographic information was collected on participants’ sexual partners, and couch-surfing hosts were not identified. As a result, disentangling the multiple roles that couch-surfing hosts may hold in a young person’s network and the extent to which their age may be associated with facilitating or buffering against sexual risk behavior among YAEH remains an area for further investigation.

Limitations concerning other measures of interest also signal opportunities for future research on HIV-related sexual risk behavior among YAEH. Measures implemented by the parent study were relatively broad in their assessment of the relationship between young adults and their sexual partner(s) and the temporality of their sexual encounters; participants were only asked to indicate their number of sexual partners in the past 30 days. Given the evidence for the influence of these relational factors on HIV risk among other populations [65–67], future research among YAEH ought to consider collecting greater detail on individual sexual partners. One proposed classification of sexual partner types includes the *established partner*,* new partner*,* occasional partner*,* one-off partner*, and *sex worker* [66]. Together with the timing of sexual encounters, such a classification can enable a more granular exploration of the types of sexual partners with whom young people are engaged and their temporal patterning, leading to more nuanced understandings of sexual risk among YAEH and opportunities to improve HIV prevention efforts.

While HIV risk among YYAEH is argued to largely be the result of higher-risk sexual activity [6], how sexual behavior among this population intersects with substance use to affect HIV risk remains a relevant area of further research [68]. Measures of substance use in the parent study broadly assessed whether participants or their sexual partner(s) were under the influence of drugs or alcohol during sex, a behavior that was observed at nearly twice the rate among unsheltered young adults compared to those who were couch-surfing or residing in shelter. However, the current study is unable to determine the nature of this substance use or assess whether specific *types* of substance use influence sexual risk. Future research on sexual behavior among YAEH should include measures on the type of substance(s) used, young people’s perceptions of this substance use, and distinguish between substance use of the participant and their sexual partner(s).

As couch-surfing was not an intended focus of the parent study, the current study is also limited by its measure of couch-surfing. Eligibility criteria required participants to be receiving services from one of the partnering drop-in centers, which in turn required young adults to be experiencing homelessness or housing insecurity in order to receive those services. It is possible that some participants were accessing certain supports at the drop-in center while transitioning into stable housing, but it is unknown how prevalent this practice is among couch-surfers. An analysis of responses to additional survey items among participants coded as *couch-surfing* indicated that endorsement of residing with another person coincided both with the duration of the participant’s homelessness and their connection to the drop-in center. While an imperfect operationalization, study findings underscore that even using a broader proxy measure, couch-surfing appears to pose distinct risks for YAEH. There is great need for a clearer conceptualization of couch-surfing and improved methods for measuring this form of homelessness among youth and young adults. In the meantime, researchers are encouraged to incorporate measures of couch-surfing that include additional qualifiers to survey response options determining a young person’s homeless situation, e.g., 'couch-surfing or temporarily residing with others *because I do not have a safe or stable place to stay'* [27], and/or additional items that assess the perceived stability of the housing arrangement [69]. 

Finally, the recruitment of participants through drop-in centers must also be considered a potential source of sampling bias, as the couch-surfing young adults in the current sample were likely underrepresented relative to their prevalence in the overall population of YAEH. Additionally, these participants likely represent a narrower range of couch-surfing experiences. While drop-in centers serve as critical safe havens to help meet the basic needs of YYAEH [70], couch-surfing young adults may be less connected to the mainstream homeless services system [11]. This may be attributed to couch-surfers’ lack of knowledge of available resources, negative experiences with or perceptions of social services, and/or being considered lower priority by both the homeless services system and themselves [11, 71]. Additionally, urbanicity may be a factor in the prevalence of couch-surfing among young adults [10]. Study participants were recruited from drop-in centers located in the two primary hubs for homeless youth services in Los Angeles, Hollywood and Venice, where couch-surfing young adults may have been less likely to be encountered relative to more suburban or rural communities.

## Conclusion

This study provides new empirical evidence that among YAEH, couch-surfing is significantly associated with increased odds of transactional sex. Findings underscore the need to better understand and attend to the unique socioenvironmental contexts of couch-surfing that may impact young people’s HIV risk-related sexual behavior—and to pursue policy changes that respond to the realities of couch-surfing among YAEH. Independent of specific host dynamics, study findings suggest that couch-surfing *in and of itself* may pose a greater risk to young people’s sexual health, safety, and well-being relative to residing in emergency shelter or transitional housing programs. This runs counter to prevailing narratives that characterize couch-surfing as a safer, less vulnerable form of homelessness. Service providers must be equipped to recognize *and* respond to young people whose couch-surfing may be intertwined with transactional sex. Engaging YAEH in assessing their level of sexual risk while couch-surfing and identifying strategies to reduce their risk for HIV and other sexually transmitted infections are vital to promoting their sexual health and well-being. While these strategies may include easy access to condoms, HIV testing, and PrEP, greater access to structural interventions that help a young person meet their basic needs are also needed. This requires a re-examination how couch-surfing youth and young adults are perceived and prioritized within mainstream homelessness response systems, and advocating for more avenues toward safe, stable, and affordable housing for young people.

## Footnotes

1 In this paper, *youth* refers to individuals under the age of 18 and *young adults* refers to individuals between the ages of 18 and 25.

2 While this measure was consistent with how couch-surfing has been assessed in earlier studies with young adults at drop-in centers [41, 42], the perceived stability of these living arrangements is unknown. It is important to note that the eligibility criteria for drop-in centers where participants were recruited emphasized that young people must be experiencing homelessness or housing insecurity to receive services. The potential shortcomings of this approach and considerations for future measures of couch-surfing are discussed further under *Limitations.*
